# Pediatric Oncology as the Next Global Child Health Priority: The Need for National Childhood Cancer Strategies in Low- and Middle-Income Countries

**DOI:** 10.1371/journal.pmed.1001656

**Published:** 2014-06-17

**Authors:** Sumit Gupta, Roberto Rivera-Luna, Raul C. Ribeiro, Scott C. Howard

**Affiliations:** 1 Division of Paediatric Haematology/Oncology, Hospital for Sick Children, Toronto, Ontario, Canada; 2 Department of Paediatrics, Faculty of Medicine, University of Toronto, Toronto, Ontario, Canada; 3 Division of Pediatric Hematology/Oncology, National Institute of Pediatrics (NIP), Mexico City, Mexico; 4 Division of Pediatric Hematology/Oncology, St. Jude Children's Research Hospital, Memphis, Tennessee, United States of America

## Abstract

Dr. Sumit Gupta and colleagues discuss the need for national cancer strategies for children in low- and middle-income countries and suggest how such strategies could be implemented.

*Please see later in the article for the Editors' Summary*

**Summary Points**

- As is already the case in high-income countries, cancer represents the leading cause of non-accidental death among children in a growing number of middle-income countries
- Meaningful declines in global childhood cancer mortality will require moving beyond the current situation through the establishment of national childhood cancer strategies
- Key components of such strategies include financial coverage, accreditation of childhood cancer centers, mandatory childhood cancer reporting and registration, development of national standards of care, and the creation of national childhood cancer governing bodies
- Challenges to implementing such strategies include a paucity of implementation research, formal policy evaluation, and costing data
- The ideal structure of such strategies in low-income countries is currently unknown, given severe resource constraints, deficits in infrastructure, and competing health needs

## Introduction

While the last several decades have witnessed tremendous advances in cure rates for childhood cancer, these improvements have not translated to low-and-middle-income countries (LMICs), where the majority of children reside [1]. In this article, we outline why pediatric cancer should now be considered a global child health priority, describe the need for national childhood cancer strategies (NCCS), and highlight necessary policy components to reduce LMIC pediatric cancer mortality rates.

## Pediatric Cancer as a Global Child Health Priority

Major shifts in the magnitude and causes of childhood mortality have occurred in many LMICs, including 106 countries with accelerated declines in childhood mortality from 1990 to 2011; 80% of this decline was due to reductions in death from infectious causes [2]. A large, and growing, proportion of global childhood mortality is therefore due to non-communicable disease [3],[4]. Indeed, 6.0% and 18.6% of deaths among children ages 5 to 14 years in lower- and upper-middle-income countries (MICs), respectively, are due to cancer [5]. As is already the case in high-income countries (HICs), cancer represents the leading cause of non-accidental death among children in a growing number of MICs [6],[7]. In absolute terms, of the 175,000 children diagnosed with cancer annually, an estimated 150,000 live in LMICs [5]. Even this figure represents a substantial underestimate given the endemic under-diagnosis and under-registration of LMIC children with cancer [1].

Unlike many adult malignancies, most pediatric cancers are not associated with modifiable risk factors and are not amenable to population-based screening and prevention programs [8]. Decreasing childhood cancer mortality thus requires accurate diagnosis followed by effective treatment. Fortunately, such treatment exists; in HICs over 80% of children with malignancies are cured [6],[9],[10]. Even simple, low-intensity treatment regimens can cure a significant portion of patients. About half of children with Burkitt lymphoma, the most common childhood malignancy in parts of sub-Saharan Africa, are curable with three to six doses of single-agent cyclophosphamide, demonstrating the achievements possible in even the most resource-limited settings [11]. Preliminary evidence suggests that such treatment is very cost effective [12].

In HICs, the dominant paradigm is to deliver pediatric cancer treatment through a limited number of treatment centers (and associated satellites) in which resources and expertise are concentrated. By contrast, in the majority of LMICs, care is currently delivered without any overarching structure or policy. Though centers of excellence exist in many LMICs, and some benefit from “twinning” partnerships with HIC centers [13]–[15], the absence of explicit national pediatric strategies results in a lack of access to care for the vast majority of LMIC children with cancer [5],[16].

## Building National Childhood Cancer Strategies

Meaningful declines in global childhood cancer mortality will require moving beyond the current situation through the establishment of NCCS. For maximal impact, NCCS should include several key policy components, as outlined in Box 1 and detailed below. While examples of such strategies are rare in LMICs, notable exceptions include the recent expansion of Seguro Popular in Mexico, which is used as an illustration throughout this article [17]–[19].

**Box 1. Key Components of National Childhood Cancer Strategies in Low- and Middle-Income Countries**

1. Financial coverage of childhood cancer treatment – Limiting financial burdens on caregivers is essential to increasing access to lifesaving therapies.
2. Accreditation of childhood cancer centers – Treating institutions should be accredited based on infrastructure, patient volumes, and reporting ability. Financial incentives may be tied to accreditation, and assistance given to centers wishing to achieve accreditation standards.
3. Mandatory childhood cancer reporting and registration – Childhood cancer registries should be created in order to allow for informed resource allocation and the evaluation of childhood cancer policy implementation.
4. Development of national standards of care – National treatment protocols should be developed which take into account local capabilities and realities. Financial incentives may be tied to the use of such protocols.
5. Creation of a national childhood cancer governing body – A multidisciplinary body should be created and tasked with monitoring the above policy components as well as with ongoing policy evaluation.

### Financial Coverage of Childhood Cancer Treatment

Effective pediatric cancer control requires financial support for families without adequate resources or private health insurance [16]. In jurisdictions with nascent universal health care systems, financial support may be accomplished through the expansion of such systems to include childhood malignancies. Policymakers may begin by covering specific malignancies depending on prevalence and available financial resources. When prioritizing financial coverage in a specific setting, other considerations may include the level of supportive care necessary during treatment, achievable cure rates, and the need for other treatment modalities, including surgery and radiation.

In Mexico, a Fund for Protection against Catastrophic Expenditures began to cover childhood acute lymphoblastic leukemia (ALL) in 2006 and all other pediatric malignancies in 2008 [18],[20]. Current efforts in China to build comprehensive insurance programs that also cover childhood cancer hold great promise, but are still in their infancy. Financial coverage, though crucial, is by itself insufficient for maximal impact, and can even create perverse incentives when regulatory and monitoring infrastructure is insufficient.

Financial incentives must be carefully structured to encourage the most desirable outcomes. Lump sum payments per patient diagnosed, as was initially done in Mexico, provided no incentive to reduce the incidence of relapse, abandonment of therapy, or toxic death. This misalignment of incentives may partially explain why rates of toxic death remained high even after health insurance was expanded [20]. Dividing remuneration into smaller sums payable as a patient reaches each treatment phase may represent a solution. However, the effectiveness and cost-effectiveness of such strategies have not been evaluated.

### Accreditation of Childhood Cancer Centers

In the absence of organized health care systems, childhood cancer treatment may be provided in a single jurisdiction by an extraordinarily high number of centers of varying quality. For example, in Colombia, pediatric oncology care for the estimated 1,800 children diagnosed with cancer each year is currently provided by 165 centers. Many centers lack sufficient expertise, resources, or patients. Expanding financial coverage may exacerbate this phenomenon, as individuals or small institutions begin to treat pediatric oncology patients for monetary gain. Financial remuneration should therefore be provided only to centers accredited on the basis of available infrastructure, patient volume, the ability to report outcomes, and the presence of trained oncology staff members, including at least one certified pediatric oncologist, pediatric oncology nurses, and other members of the multidisciplinary team. By 2009, 47 Mexican hospitals had been certified by the Ministry of Health in this way, and were therefore eligible to receive financial compensation [21]. Ideally, regional referral systems allowing more complicated cases to be treated in highly specialized institutions would also be developed.

### Mandatory Childhood Cancer Reporting and Registration

In 2006, only 4% of Asian populations and 1% of sub-Saharan African populations were covered by high-quality population-based cancer registries [22]. Though the effort required to build such registries is considerable, the establishment of childhood cancer population-based registries may be more feasible than adult registries, since the number of pediatric patients is a small fraction of the total cancer cases. Efforts to build such pediatric registries are currently underway in Guatemala and El Salvador. These registries can evolve from hospital-based registries that have already undergone the necessary processes to ensure accurate data collection, as demonstrated in Argentina [23].

Such registries, when combined with mandatory reporting of childhood cancer cases, are integral to national strategies for several reasons. Population-based registries document the burden of disease, allowing for informed resource planning and allocation. Collecting outcome data also allows for the impact of childhood cancer policies to be evaluated. Finally, the documentation of where childhood cancer patients are treated will identify centers with poor outcomes, which can then be eligible for increased resources, corrective action, or in extreme cases, closure.

### Development of National Standards of Care

One of the achievements of pediatric oncology in recent decades is the refinement of risk stratification systems, allowing for an assessment of the aggressiveness of a particular child's cancer and for treatment intensity to be matched to disease risk, thus reducing both under-treatment and over-treatment [24],[25]. Avoiding overtreatment is crucial in LMICs, since it carries with it an increased risk of toxic death from causes such as infection and hemorrhage [26],[27]. At some point, any benefit in disease control by intensifying treatment will be outweighed by an increase in toxicity. Finding the balance point for each malignancy at each pediatric cancer center is key to optimizing therapy and curing the maximum number of children possible. This ideal point depends not only upon the malignancy in question, but also upon a particular center's ability to provide supportive care to prevent and manage treatment complications [27],[28].

In many LMIC centers, supportive care capabilities lag behind those in HICs. Implementing unmodified treatment protocols designed for HICs in LMIC centers where supportive care is less developed therefore inevitably leads to high levels of toxicity [27]. For example, in the Dominican Republic, a relatively intensive HIC treatment regimen was used in 91 children with acute lymphoblastic leukemia (ALL) between 2005–2007, resulting in a 2-year survival of 40% and a toxic death rate of 32%. Decreasing treatment intensity improved overall 2-year survival to 70% and decreased the toxic death rate to 8% [28].

National treatment protocols that have been designed and adapted to local circumstances by appropriate experts are therefore integral to maximizing cure rates. In Mexico, the use of such protocols was mandatory, and tied to the accreditation process [20].

### Creation of a National Childhood Cancer Governing Body

Finally, a body with the ultimate responsibility of creating and implementing the national strategy is required. This committee should include clinicians, epidemiologists, and policymakers, and be either part of, or associated with, the Ministry of Health. Responsibilities of the body would include accrediting treatment centers, developing and updating national standards of care, planning new infrastructural capacity in jurisdictions with need, workforce training and support, ensuring stable drug supplies, and ongoing policy evaluation.

## Challenges to Implementation

### Policy Evaluation

A major challenge to the implementation of NCCS is the absence of formal impact assessments. While the principles outlined above are based in sound theory and extensive practical experience, rigorous policy evaluation is lacking. Indeed, preliminary results of the impact of Seguro Popular in Mexico are mixed. While the proportion of eligible cases funded by the Fund for Protection against Catastrophic Expenditures increased from 3.3% to 55.3% between 2006 and 2009, the overall number of children treated did not seem to change [20],[21]. The shifting of cost burdens from families to government may simply precede increases in the number of patients treated, though this has not yet been proven. Though rates of treatment abandonment decreased and overall cure rates increased over the same time period, whether this was directly due to improved financial coverage is unknown. Opportunity and ancillary costs (e.g., travel) remain significant caregiver burdens that may explain persistent regional inequalities [20],[21]. Indeed, such costs are a significant burden even in HICs with universal health care systems, and are likely to play an even larger role in LMICs [29]. Finally, the outcomes of most children treated outside Seguro Popular institutions remain unknown. As new strategies are realized in additional jurisdictions, formal policy evaluation must be incorporated.

### Cost and Cost-Effectiveness Data

**Figure 1:**
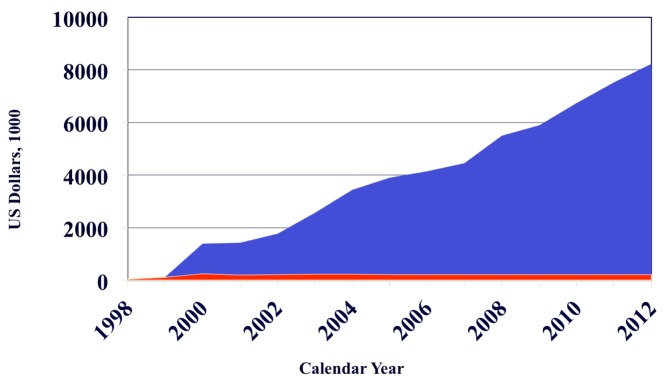
Funding sources of the Unidad Nacional de Oncologia Pediátrica of Guatemala. The red area indicates funding from St. Jude Children's Research Hospital and the blue area funding from all other sources. An initial outlay of funds from St. Jude was subsequently leveraged into additional resources from both government and private donors. The creation of an independent fundraising organization (Fundación Ayúdame a Vivir, http://ayuvi.org.gt) was essential to this outcome. Similar successes may be possible in other settings; the role and best use of seed funding from HIC centers requires further investigation.

Second, the cost of implementing such policies has not been described. While childhood cancer treatment is likely to be highly cost-effective given the large number of potential years of life saved [12], rigorous costing studies are required to better inform health policymakers. It is worth noting that pediatric oncology may represent a unique funding model in its ability to attract private resources that would otherwise have remained outside the health sector. For example, in Guatemala an initial outlay of funds from an HIC twinning partner was subsequently leveraged into additional resources from both government and private donors (Figure 1). Similar success may be possible in other settings.

### Implementation Research

**Figure 2:**
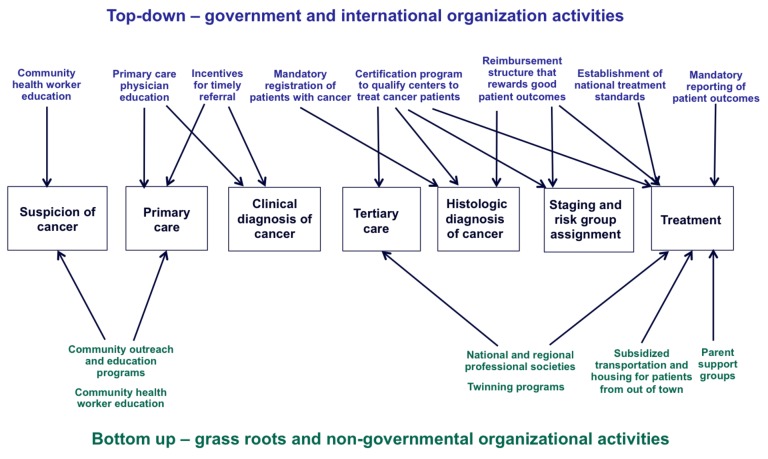
Top-down and bottom-up approaches to improve pediatric cancer care.

Third, implementation research is required. Descriptions of how NCCS came to be seen as priorities by policymakers and governmental officials in Chile, Mexico, and China would be of tremendous use to other LMICs. To date, advocacy by grassroots nongovernmental organizations, caregivers, and local medical leaders have been responsible for the creation of childhood cancer policies, as opposed to directives from national and supra-national agencies such as the World Health Organization. Ideally, top-down and bottom-up efforts would be coordinated and integrated to achieve maximal impact (Figure 2).

### Low-Income Countries

Many of the above policy recommendations are best suited to and most easily implemented in MICs. Low-income countries (LICs) face additional challenges, given severely constrained resources, limited infrastructure, and significant competing health concerns. Nonetheless, several examples of successful childhood cancer treatment in LICs exist [11],[15]. How health policy can support such LIC efforts while still taking into account LIC-specific realities should be the focus of health policy and health economics research efforts.

### Correcting Misconceptions

Finally, and perhaps most importantly, many laypeople, policymakers, and decision makers may see childhood cancer treatment as far too complex and costly for most LMICs. Currently, most private and public international funds are directed towards adult cancer. It is worth remembering however that the successful control of HIV was also seen as tremendously complex and costly, and therefore beyond the capabilities of most LMIC health systems. Through international coordination of advocacy, research, and policy, the latest report on HIV/AIDS concluded that “remarkable increases in access to life-saving antiretroviral therapy” continued to be seen, though substantial effort was of course still required [30]. We believe that similar achievements are feasible for children with cancer in LMICs.

## Conclusions

Pediatric malignancies account for a growing proportion of overall global childhood mortality, justifying renewed efforts to improve cure rates for this population in resource-limited settings. NCCS offer the best hope of reaching this goal, as summarized in Box 2. While the financial coverage of pediatric oncology care should be an integral part of any such strategy, maximal impact will require additional policies addressing the structural aspects of care delivery and the creation of childhood cancer registries.

**Box 2. Key Recommendations**

1. National childhood cancer strategies should be designed and implemented in LMICs, with key components as outlined in the text and in Box 1.
2. The implementation and impact of existent national strategies in LMICs should be evaluated; new national strategies should include an evaluation component.
3. Additional data on the cost and cost-effectiveness of such strategies are needed.
4. Health policy and health economics research is required to determine how to best adapt these recommendations to account for the additional challenges inherent to LICs.

## Abbreviations

HIC: high-income country
LIC: low-income country
LMIC: low- and middle-income countries
MIC: middle-income country
NCCS: national childhood cancer strategies
